# Confidence in Prison Bars

**Published:** 1874-12

**Authors:** 


Confidence in Prison Bars. — A new teacher at the Springfield City Farm Truant School is dealing with the boys on the "confidence system," and thus far with success. She discards bars and bolts, puts the boys upon their "honor," and lets them run. No one has abused it and ran away yet, and she thinks if one should try it, the rest would run after him and bring him back. So strong is her confidence in her boys, that she recently sent one of them down to the City Treasurer's Office to draw her salary, with instructions to deposit it for her in the savings bank. The errand was promptly and correctly done, and the boy was back in his quarters in due time. The doubters don't believe in the system, but Miss Bascom does, and, so far, the boys do also.

